# Endoscopic resection of a giant broad-based gastric lipoma in an adolescent: a case report and literature review

**DOI:** 10.1186/s12887-025-05736-z

**Published:** 2025-05-15

**Authors:** Shaojun Wang, Hong Yang, Na Jiang, Shengbo Jin

**Affiliations:** 1 Department of Gastroenterology, The Affiliated Hospital of Qingdao University, Qingdao, 26600 China; 2 Emergency Department, The Affiliated Hospital of Qingdao University, Qingdao, 26600 China

**Keywords:** Gastric lipomas, Giant, Endoscopic submucosal dissection, Broad-based, Case report

## Abstract

Gastric lipomas are rare benign gastric tumors, with pediatric and adolescent cases being exceptionally uncommon. This report describes a case of a 17-year-old girl with a giant gastric lipoma (> 8 cm) and the first successfully treated via endoscopic submucosal dissection (ESD). This case demonstrates that minimally invasive therapy can serve as an alternative to surgery for giant gastric lipomas in rare clinical scenarios.

**Supplementary Information:**

The online version contains supplementary material available at 10.1186/s12887-025-05736-z.

## Introduction

Gastric lipomas are rare benign tumors, accounting for less than 1% of all gastric neoplasms [1]. They are typically slow-growing and more common in adults, with large lipomas (> 4 cm) being exceptionally rare [2]. Most small gastric lipomas are asymptomatic and managed conservatively. However, larger lesions may cause complications such as abdominal pain, luminal obstruction, gastrointestinal bleeding, ulceration, gastric outlet obstruction with vomiting, or even rare intussusception, necessitating intervention [3, 4]. The management of large gastric lipomas remains challenging due to the lack of standardized guidelines. This decision often depends on factors such as tumor size, location, and morphology, as well as the patient’s clinical status. Traditional management relies on surgical resection, though endoscopic resection has emerged as a minimally invasive alternative in adults [5, 6]. However, endoscopic management in pediatric and adolescent patients remains undocumented, with no prior reports of technical success in this demographic. Here, we report the first case of endoscopic treatment for a giant, broad-based gastric lipoma (> 8 cm) in an adolescent and discusses its feasibility in conjunction with the literature.

## Case report

**Fig. 1:**
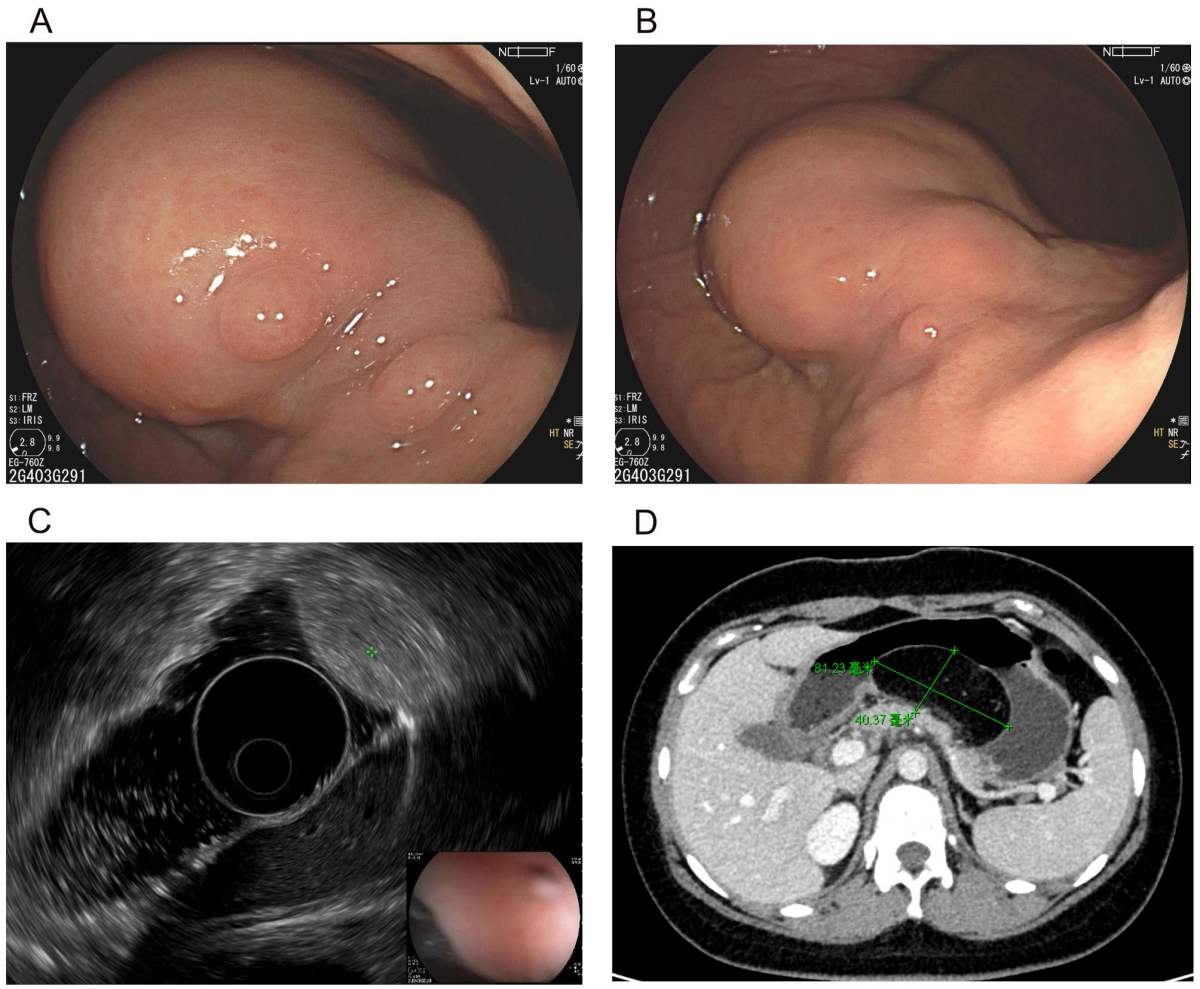
A large, smooth, soft mass in the stomach’s body can obstruct the lumen when the stomach is not fully expanded (**A**). When inflated, the mass appears to have a broad base (**B**). EUS demonstrates uniform high echogenicity (**C**), and CT reveals an 8.12 cm fatty density within the wall of the gastric body (**D**)

A 17-year-old girl presented with a 3-month history of progressive postprandial abdominal distension and indigestion, accompanied by intermittent vomiting (1–2 episodes weekly). Physical examination was unremarkable, and there was no significant family history of note. Initial gastroscopy revealed a large, broad-based, smooth-surfaced soft mass (7 × 8 cm) in the gastric body, completely obstructing the lumen during non-distended phases. Subsequent endoscopic ultrasound (EUS) demonstrated a hyperechoic submucosal lesion with well-defined margins and no infiltrative features, consistent with a benign lipoma. To further characterize the lesion and exclude malignancy, contrast-enhanced abdominal CT was performed, revealing a homogeneous fat-density mass confined to the gastric wall, without evidence of lymphadenopathy or metastatic disease (Fig. 1).

During a preoperative multidisciplinary discussion involving gastrointestinal surgeons and anesthesiologists, the decision to proceed with endoscopic submucosal dissection (ESD) was made based on several factors. First, the patient, a young female, strongly desired to preserve full gastric function and avoid abdominal scarring. Second, preoperative imaging (CT, EUS, and gastroscopy) indicated that the tumor originated from the submucosal layer, protruded into the lumen, and showed no evidence of lymph node involvement or deep infiltration. Additionally, the lesion’s location was favorable for endoscopic manipulation.

**Fig. 2:**
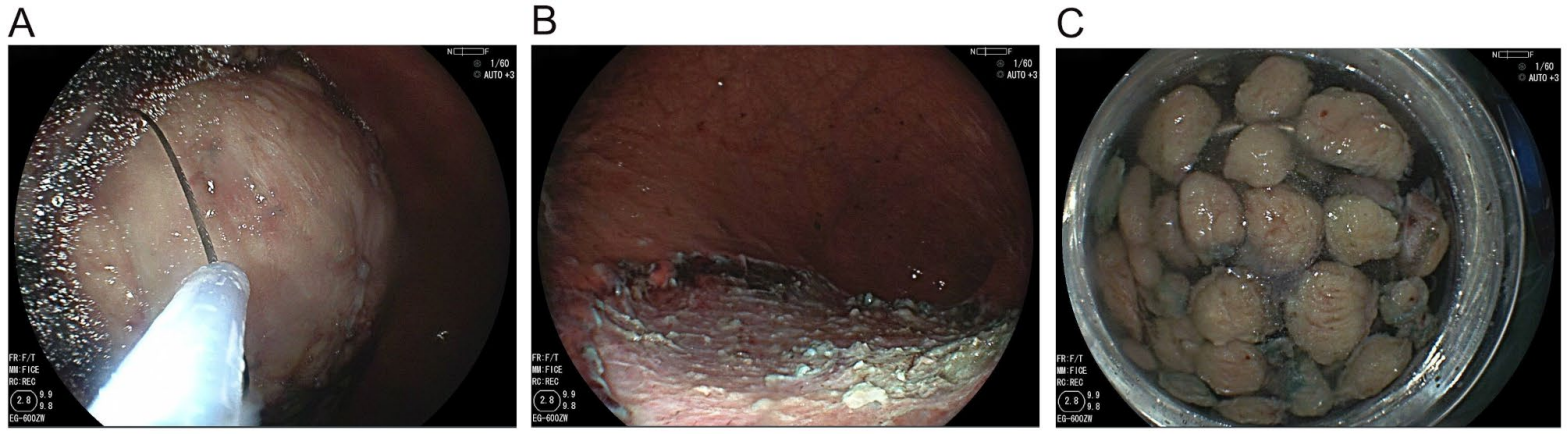
The mass was completely excised (**A**), fragmented into small pieces (**C**), and the wound bed was clean (**B**)

During the ESD procedure, methylene blue saline was injected into the submucosal layer to facilitate dissection. The lesion was carefully dissected, with particular attention given to managing blood vessels in the submucosal layer. Challenges such as bleeding and visual obstruction caused by fat tissue cauterization were successfully overcome, and the tumor was completely removed without perforation. Due to the large size of the excised mass, it could not be retrieved intact through the esophagus. Instead, the mass was fragmented into approximately 30 pieces, each measuring 2–3 cm in diameter, using snares and electrocoagulation. These fragments were then removed orally in multiple passes (Fig. 2).

**Fig. 3:**
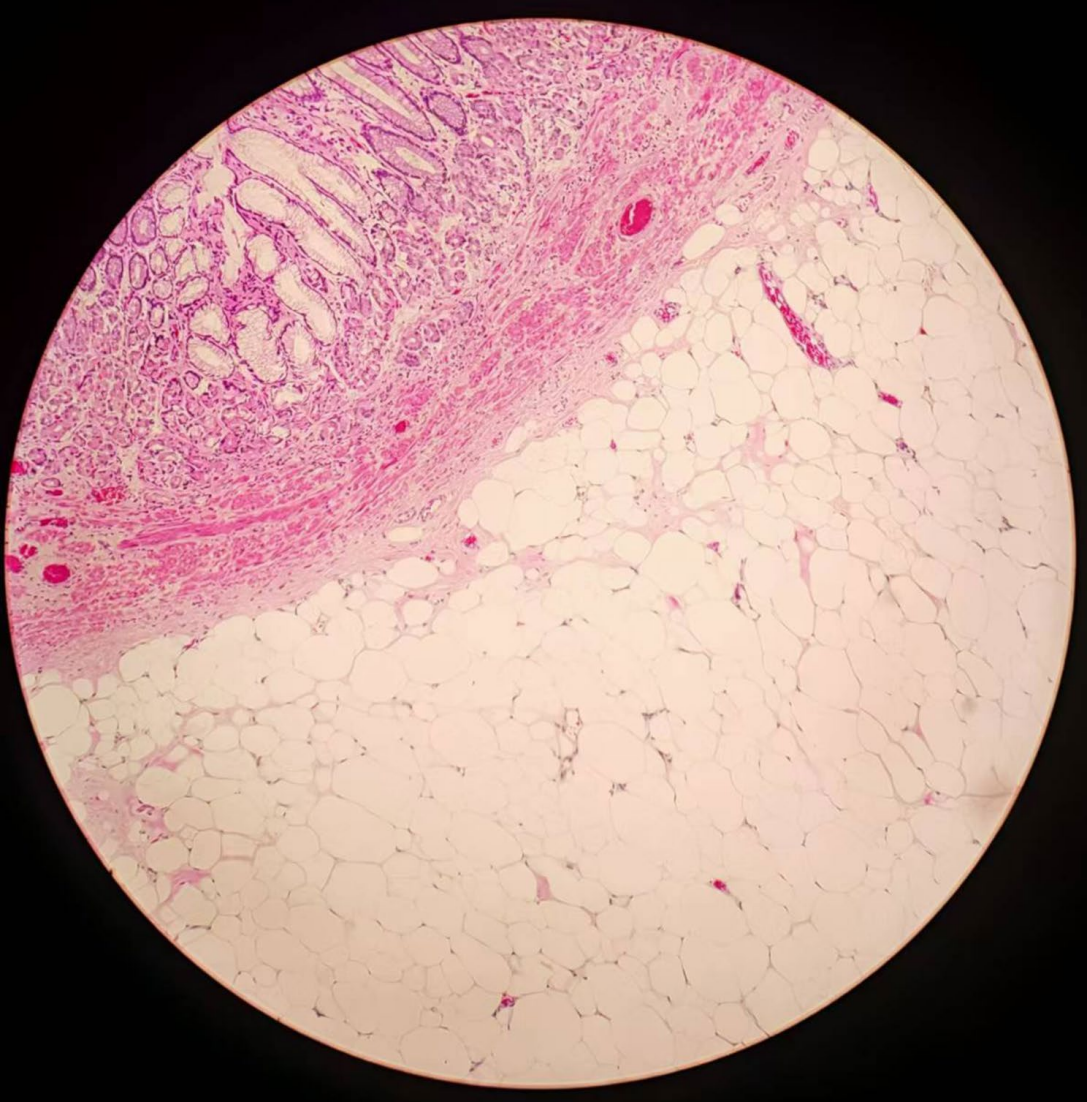
H&E staining (100×) demonstrating mature adipose tissue with minimal stroma, consistent with gastric lipoma

Postoperatively, the patient’s wound did not perforate, but due to its large size, it could not be closed with titanium clips. The patient was monitored for 72 h with serial abdominal and vital sign assessments. No clinical signs of perforation (e.g., fever, peritoneal irritation) were observed, and per institutional protocols, contrast imaging was not performed. Oral feeding was resumed gradually, and the patient recovered well without complications, ultimately discharged in stable condition. Histopathological examination confirmed the diagnosis of a benign gastric lipoma (Fig. 3).

This case demonstrates the potential feasibility of endoscopic en bloc resection for a giant gastric lipoma (>8 cm) in an adolescent. The procedure avoided gastrectomy, and preliminary outcomes suggest preserved gastric function with no immediate complications. Patient and family satisfaction was documented, though long-term follow-up remains essential to evaluate recurrence risks and functional stability.

## Discussion

Gastric lipomas are rare benign tumors that mainly affect adults over the age of 50, with only a few clinical reports of cases in pediatric and adolescents [7]. This case is distinguished by its occurrence in a 17-year-old female and the exceptionally large tumor size (> 8 cm), marking the first successful endoscopic resection of a giant broad-based gastric lipoma in this demographic.

A systematic review of 32 giant gastric lipomas (> 4 cm) identified only one prior endoscopic resection success in adults. In pediatric populations, endoscopic attempts have consistently failed, exemplified by a 13-year-old male whose resection was abandoned due to procedural pain and broad-base morphology, ultimately requiring surgery [2]. Our case, the seventh reported giant gastric lipoma in patients ≤ 18 years old, challenges this historical precedent by demonstrating the feasibility of endoscopic submucosal dissection (ESD) in rigorously selected adolescents (Table 1).

**Table 1:** Reported giant gastric lipoma cases (≤ 18 years Old)

Case	Publication year	Age(years)/Sex	Size (cm)	Location	Symptoms	Treatment
1	1966 [8]	12/F	NS	Stomach	Hemorrhage	NS
2	1988 [9]	2/M	NS (causing obstruction)	Antrum	Vomiting, gastro-duodenal intussusception	Emergency laparotomy
3	1997 [10]	13/M	8 × 4 × 3	Stomach	Hematemesis, melena, abdominal pain	Failed endoscopic resection due to broad base and pain→ Surgery
4	1999 [11]	11/F	Multiple, NS	Body/antrum	Abdominal pain	Follow-up due to mild symptoms
5	2007 [12]	10/M	4	Antrum	Epigastric pain, melena	Open surgery
6	2010 [7]	12/M	12 × 8	Stomach	Hematemesis, melena	Open surgery

NS: not specified; F: female; M: male

For small, asymptomatic lipomas, observational strategies with periodic endoscopic surveillance are appropriate. Pharmacological therapy (e.g., proton pump inhibitors, mucosal protectants) may alleviate symptoms such as dyspepsia or epigastric pain but does not target the lesion itself. For larger, symptomatic, or rapidly growing lipomas, endoscopic or surgical resection is indicated. While open or laparoscopic surgery remains conventional for giant lesions, endoscopic resection has demonstrated comparable efficacy for tumors ≤ 6 cm, with similar recurrence rates and survival outcomes to surgical approaches [13, 14]. However, consensus guidelines are lacking, particularly for giant lipomas (> 5 cm). Existing case reports of endoscopic resection for tumors 5–10 cm predominantly involve pedunculated or subpedunculated lesions without deep mural invasion, whereas broad-based tumors, as in this case, often necessitate surgery [2, 4, 5].

Another unique aspect of this case is the broad-based morphology, which increases technical complexity due to the absence of a pedicle. Preoperative contrast-enhanced CT and EUS confirmed submucosal origin with intraluminal growth, no lymph node involvement, and favorable anatomical positioning, enabling complete endoscopic resection.

Endoscopic resection of giant gastric lipomas poses unique challenges, particularly when fragmented extraction is required for large lesions, as tumor implantation risks necessitate preoperative confirmation of benign histology. While homogeneous fat density on CT and hyperechoic submucosal features on EUS strongly suggested a benign lipoma, imaging alone cannot exclude rare mimics like well-differentiated liposarcoma. EUS-guided biopsy remains critical to confirm benignity and prevent tumor seeding risks. In this case, biopsy omission was justified by definitive imaging, the patient’s young age without sarcoma risk factors, and multidisciplinary consensus prioritizing minimally invasive resection. Postoperative histopathology confirmed benignity, validating our approach. However, we emphasize that preoperative biopsy remains the gold standard for submucosal lesions, and its omission here reflects a limitation. This case highlights the necessity of balancing innovation with safety through meticulous imaging, multidisciplinary collaboration, and transparent patient-family counseling when deviating from standard protocols.

The extensive submucosal dissection required for this broad-based lesion inherently carries a risk of perforation. While our patient recovered without complications, postoperative contrast imaging is a prudent measure to exclude occult perforation, particularly in cases involving large or complex resections. Future protocols should incorporate such imaging to enhance safety, even in asymptomatic patients.

This case holds clinical significance as one of the few reported giant gastric lipomas in young patients and the first demonstrating endoscopic feasibility for a broad-based lesion. It underscores the importance of individualized treatment planning, emphasizing preoperative imaging (CT/EUS), lesion characteristics (benign, non-infiltrative), and operator expertise. this report advocates for expanding minimally invasive strategies in pediatric gastroenterology, particularly to reduce surgical morbidity and preserve gastric function in adolescents. However, as a single case report, long-term outcomes remain unverified, and success depends on operator expertise and favorable tumor characteristics (benign, non-infiltrative). Further validation in larger studies is necessary, particularly for adolescents with complex lesions.

## Electronic supplementary material

Below is the link to the electronic supplementary material.

Supplementary Material 1

## Data Availability

No datasets were generated or analysed during the current study.
