# Supplementary material for: Endoscopic resection of a giant broad-based gastric lipoma in an adolescent: a case report and literature review
Source: BMC Pediatr. 2025 May 15;25:388. doi: 10.1186/s12887-025-05736-z (PMC12079961; doi:10.1186/s12887-025-05736-z)
Supplement: Supplementary file 1 — Supplementary Material 1 [file 12887_2025_5736_MOESM1_ESM.docx]

**Submission ID 4289f5ea-c82f-48d0-b663-01f279098ba9**

**Endoscopic Resection of a Giant Broad-Based Gastric Lipoma in an Adolescent: A Case Report and Literature Review**

**CARE Checklist of information to include when writing a case report**

| **Topic** | **Item** | **Checklist item description** | **Reported on Line** |
| --- | --- | --- | --- |
| **Title** | **1** | The diagnosis or intervention of primary focus followed by the words “case report” | Line 28-29 |
| **Key Words** | **2** | 2 to 5 key words that identify diagnoses or interventions in this case report, including "case report" | Line 39-40 |
| **Abstract**  **(no references)** | **3a** | Introduction: What is unique about this case and what does it add to the scientific literature? | Line 33-35 |
|  | **3b** | Main symptoms and/or important clinical findings | Line 32-33 |
|  | **3c** | The main diagnoses, therapeutic interventions, and outcomes | Line 34-35 |
|  | **3d** | Conclusion—What is the main “take-away” lesson(s) from this case? | Line 35-37 |
| **Introduction** | **4** | One or two paragraphs summarizing why this case is unique (**may include references**) | Line 44-58 |
| **Patient Information** | **5a** | De-identified patient specific information | Line 62 |
|  | **5b** | Primary concerns and symptoms of the patient. | Line 62 |
|  | **5c** | Medical, family, and psycho-social history including relevant genetic information | Line 63 |
|  | **5d** | Relevant past interventions with outcomes | N/A |
| **Clinical Findings** | **6** | Describe significant physical examination (PE) and important clinical findings | Line 62-63 |
| **Timeline** | **7** | Historical and current information from this episode of care organized as a timeline | N/A |
| **Diagnostic**  **Assessment** | **8a** | Diagnostic testing (such as PE, laboratory testing, imaging, surveys) | Line 64-69 |
|  | **8b** | Diagnostic challenges (such as access to testing, financial, or cultural) | Line 64-69 |
|  | **8c** | Diagnosis (including other diagnoses considered) | Line 69 |
|  | **8d** | Prognosis (such as staging in oncology) where applicable | Line 103-104 |
| **Therapeutic**  **Intervention** | **9a** | Types of therapeutic intervention (such as pharmacologic, surgical, preventive, self-care) | Line 78-85 |
|  | **9b** | Administration of therapeutic intervention (such as dosage, strength, duration) | Line 86-94 |
|  | **9c** | Changes in therapeutic intervention (with rationale) | N/A |
| **Follow-up and**  **Outcomes** | **10a** | Clinician and patient-assessed outcomes (if available) | Line 102-103  Line 107-108 |
|  | **10b** | Important follow-up diagnostic and other test results | Line 103-104 |
|  | **10c** | Intervention adherence and tolerability (How was this assessed?) | Line 80-85 |
|  | **10d** | Adverse and unanticipated events | N/A |
| **Discussion** | **11a** | A scientific discussion of the strengths AND limitations associated with this case report | Line 117-121  Line 146-150  Line 161-165 |
|  | **11b** | Discussion of the relevant medical literature **with references** | Line 122-128  Line 134-145 |
|  | **11c** | The scientific rationale for any conclusions (including assessment of possible causes) | Line 146-150 |
|  | **11d** | The primary “take-away” lessons of this case report (without references) in a one paragraph conclusion | Line 151-154 |
| **Patient Perspective** | **12** | The patient should share their perspective in one to two paragraphs on the treatment(s) they received | Line 105-110 |
| **Informed Consent** | **13** | Did the patient give informed consent? Please provide if requested | **Yes √**  **No** |
